# Meeting of State Societies

**Published:** 1875-04

**Authors:** 


The Medical Association of Georgia holds its annual session the present year at Savannah, commencing on Wednesday, the 21st of April. The Mississippi State Association convenes at Vicksburg on the first Wednesday in April; the Alabama State Association at Montgomery on the second Tuesday in April. Let all who can lay aside for a few days their professional labors at home and recreate themselves at their several State meetings.

